# Plasma lncRNA GAS8-AS1 as a Potential Biomarker of Papillary Thyroid Carcinoma in Chinese Patients

**DOI:** 10.1155/2017/2645904

**Published:** 2017-07-11

**Authors:** Dongxue Zhang, Xin Liu, Bojun Wei, Guoliang Qiao, Tao Jiang, Zhenwen Chen

**Affiliations:** ^1^Department of Endocrinology, Beijing Shijitan Hospital, Capital Medical University, Beijing 100038, China; ^2^Department of Medical Genetics and Developmental Biology, Capital Medical University, Beijing 100069, China; ^3^Department of Thyroid and Neck Surgery, Beijing Chaoyang Hospital, Capital Medical University, Beijing 100020, China; ^4^Department of Medical Oncology, Beijing Shijitan Hospital, Capital Medical University, Beijing 10038, China

## Abstract

**Background:**

Long noncoding RNAs (lncRNAs) were recently shown to have potential in the diagnosis and prognosis for numerous cancers. lncRNA GAS8-AS1 is decreased in papillary thyroid cancer (PTC) tissue, but its plasma expression and clinical value in patients with PTC remain unknown.

**Materials and Methods:**

We investigated the expression profile of plasma GAS8-AS1 in 97 patients with PTC and 39 patients with nodular goiter by quantitative real-time polymerase chain reaction.

**Results:**

GAS8-AS1 expression in plasma was downregulated in patients with PTC in comparison with those in nodular goiters (*P* < 0.001). A low level of plasma GAS8-AS1 expression was correlated with lymph node metastasis (LNM) (*P* < 0.001). Multivariate analysis showed that a reduced GAS8-AS1 level in plasma was associated with LNM (*P* < 0.05). The area under the receiver operating characteristic curve for GAS8-AS1 was 0.746 in LNM prediction (*P* < 0.001).

**Conclusion:**

The present study indicates that circulating GAS8-AS1 is a potential biomarker for PTC diagnosis and LNM prediction.

## 1. Introduction

Thyroid cancer is the most prevalent endocrine malignancy, and its incidence has increased dramatically over the last several decades [1]. The incidence of thyroid cancer is 6.6/100,000 in China and is among the top 10 most common cancers in females [2]. Papillary thyroid carcinoma (PTC), named for its histopathological structure, accounts for approximately 80% of all thyroid cancers in adults. Cytopathology analysis by fine-needle aspiration biopsies (FNAB) has dramatically improved PTC diagnosis accuracy. However, there are some limitations to this method, including difficulty in sampling small tumors, inconclusive diagnosis in up to 35% of patients [3], and bleeding. Thus, biomarkers for diagnosis are needed. Although most patients with PTC have good prognosis, patients with certain clinicopathological features such as larger tumor size, lymph node metastasis (LNM), and advanced TNM stage are associated with a worse clinical outcome [4]. While the association between molecules in the thyroid (such as BRAF mutation [5, 6] and fibronectin-1 expression [7]) and aggressive features has been investigated in several studies, potential circulating biomarkers are required to estimate the aggressiveness of cancer in patients with PTC.

Long noncoding RNAs (lncRNAs) are RNA transcripts longer than 200 nucleotides without protein coding function. lncRNAs have been identified as crucial regulators in a variety of tumor growth or metastasis [8] processes by influencing the levels of various molecules, chromatin structure, transcriptional activity, mRNA stability, mRNA posttranscriptional processing, or mRNA translation. A few studies focusing on the functions of lncRNA in patients with PTC have been published. lncRNA GAS8-AS1 was found to be the second most frequently mutated gene in Chinese patients with PTC, but not in Caucasian patients. lncRNA GAS8-AS1 has a novel role in tumor suppression. lncRNA GAS8-AS1 c.713A>G/714T>C nucleotide substitution significantly reduces RNA expression. Moreover, reduced lncRNA GAS8-AS1 levels were observed in PTC tissue compared to adjacent thyroid tissues of Chinese patients [9]. The expression characteristics of lncRNA GAS8-AS1 in the plasma remain unknown.

In the current study, we found that the expression level of lncRNA GAS8-AS1 was reduced in the plasma. In addition, plasma lncRNA GAS8-AS1 was significantly correlated with cervical lymph node metastasis (LNM) and sex. lncRNA GAS8-AS1 in the plasma may be a novel biomarker for PTC in Chinese people.

## 2. Materials and Methods

### 2.1. Patients and Samples

We collected human plasma after gaining approval from the Institutional Ethics Committees of Beijing Shijitan Hospital. Ninety-seven samples and clinical data of PTC and 39 patients with nodular goiter (NG) were obtained in Beijing Shijitan Hospital between January 2015 and June 2016. All whole blood samples were collected under fasting conditions before surgery. Samples of 10 mL of blood were obtained from each patient in K2-EDTA tubes and centrifuged at 1200×g for 10 min at 4°C within 1 h of collection. All plasma samples were immediately transferred to RNase/DNase-free tubes. Next, 25 *μ*L plasma was diluted 1 : 3 in phosphate-buffered saline before spectrophotometric analysis. Samples were considered to exhibit hemolysis and were excluded when [A415 sample—A415PBS] exceeding 0.15. All samples were stored at −80°C until use. The diagnosis of PTC and nodular goiter was confirmed by two pathologists independently. The 97 patients with PTC included 26 males and 71 females with a mean age of 44.01 ± 11.15 years (24–76 years), while 39 patients with NG included 6 males and 33 females with a mean age of 50.72 ± 14.57 years (30–74 years). No patient involved in this study received any other cancer treatment preoperatively.

### 2.2. RNA Extraction

Total RNA was extracted from the plasma using Trizol LS Reagent (Invitrogen Life Technologies, Carlsbad, CA, USA) according to the manufacturer's instructions. Samples were centrifuged at 12,000×g for 10 min at 4°C to completely remove cell debris. Next, 750 *μ*L Trizol LS reagent was mixed with 250 *μ*L plasma. After vortex mixing for 30 s and keeping stationary for 5 min, 200 *μ*L chloroform was added to the tubes. The mixture was shaken vigorously by hand and incubated for 3 min at 15–30°C. Next, the sample was centrifuged at 12,000×g for 15 min at 4°C. The upper aqueous phase was transferred to a new tube, and an equal volume of isopropanol (500 *μ*L) was added to the supernatant. After mixing and incubating at room temperature for 10 min, the mixture was centrifuged at 12,000×g for 15 min at 4°C. The RNA pellet was washed with 75% ethanol (1 mL) after the removal of the supernatant. Next, the samples were subjected to vortex mixing and centrifugation at 7500×g for 5 min at 15–30°C. The pellet was dissolved in RNase-free water and incubated at 55–60°C for 10 min. Total RNA quality was confirmed using a NanoDrop® ND-1000 spectrophotometer (Thermo Fisher Scientific, Waltham, MA, USA) to ensure that the OD A260/A280 ratio was close to 2.0 (from 1.8 to 2.1). RNA integrity was evaluated with an Agilent 2100 Bioanalyzer (Agilent Technologies, Santa Clara, CA, USA).

### 2.3. RT-qPCR Detection

RT-qPCR was conducted using 2X PCR master mix (Arraystar, Rockville, MD, USA) on a ViiA 7 Real-time PCR System (Applied Biosystems, Foster City, CA, USA) following the manufacturer's instructions. The primers for PTCSC3 and *β*-actin, which was selected as a housekeeping gene, were designed and synthesized by KangChen Bio-tech (Shanghai, China). Primer sequences were as follows: F 5′GACAAGACAACGAGCAAACAAG3′ and R 5′GGAGCCTCTAAAGGTCTGTGAC3′ for lncRNA GAS8-AS1 and F 5′GTGGCCGAGGACTTTGATTG3′ and R 5′CCTGTAACAACGCATCTCATATT3′ for *β*-actin. PCR was conducted as follows: 95°C denaturation for 10 min, followed by 40 cycles at 95°C for 10 s, 60°C for 60 s, and 95°C for 10 s. The relative expression level was analyzed using the 2^-ΔΔCT^ method. Each experiment was performed in triplicate.

### 2.4. Statistical Analysis

All statistical analyses were performed by SPSS (Version 22.0 SPSS, Inc., Chicago, IL, USA). Quantitative data were expressed as the mean ± standard deviation (mean ± SD). Differences between the means were compared using Student's *t*-test. Qualitative data are shown as numbers or percentages. The data were analyzed with *χ*^2^ test. Two receiver operating characteristic curves (ROCs) were established to evaluate whether GAS8-AS1 could predict nodular malignancy and LNM. Multivariate analyses were conducted by logistic regression. The results were represented as the odds ratio (OR) and 95% confidential interval (CI). *P* < 0.05 was considered statistically significant.

## 3. Results

### 3.1. Downregulation of lncRNA GAS8-AS1 in PTC Plasma

**Figure 1:**
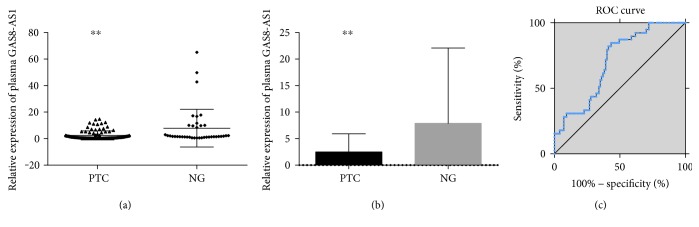
RT-qRCR and ROC curve analysis for GAS8-AS1 as a PTC diagnosis biomarker. (a) Scatter plots and (b) bar charts of plasma GAS8-AS1 levels from papillary thyroid cancer (PTC) (*n* = 97) and nodular goiter (NG) (*n* = 39). (c) ROC curve to evaluate the diagnostic performance of GAS8-AS1 (^∗∗^*P* < 0.001).

As shown in Figures 1() and 1(), GAS8-AS1 was reduced in PTC plasma samples compared to those in NG samples. Multivariate analysis showed that a reduced GAS8-AS1 level in the plasma was an independent risk factor of PTC (Table 1). ROC was also performed to evaluate the diagnostic value of GAS8-AS1 between PTC and NG (Figure 1()). The area under curve (AUC) was up to 0.702 (95%CI = 0.612–0.792, *P* < 0.001). The sensitivity and specificity were 84.62% and 56.70%, respectively, when the Youden index reached a maximum.

### 3.2. Association between lncRNA GAS8-AS1 in Plasma and Clinic-Pathological Characteristics

To explore the association between lncRNA GAS8-AS1 in plasma and clinic-pathological parameters, patients with PTC were divided into two groups according to the median level of lncRNA GAS8-AS1 (low expression and high expression). The results showed that low lncRNA GAS8-AS1 expression was associated with cervical lymph node metastasis and the male sex (*P* < 0.05). However, low lncRNA GAS8-AS1 expression was not related to age, tumor size, extrathyroidal extension, or advanced TNM stage (Table 2).

### 3.3. Correlation between Expression Level of Plasma lncRNA GAS8-AS1 and Clinic-Pathological Parameters in Papillary Thyroid Microcarcinoma (PTMC)

To further investigate the correlation between the expression level of plasma GAS8-AS1 and clinic-pathological parameters in papillary thyroid microcarcinoma (PTMC), we analyzed the data in cancers with diameters less than 1 cm (*n* = 68). Downregulated GAS8-AS1 expression was found to be significantly associated with LNM and the male sex (*P* < 0.05) in PTMC (Table 3). However, there was no correlation between GAS8-AS1 and other characteristics.

### 3.4. Relationship of Plasma lncRNA GAS8-AS1 and LNM

**Figure 2:**
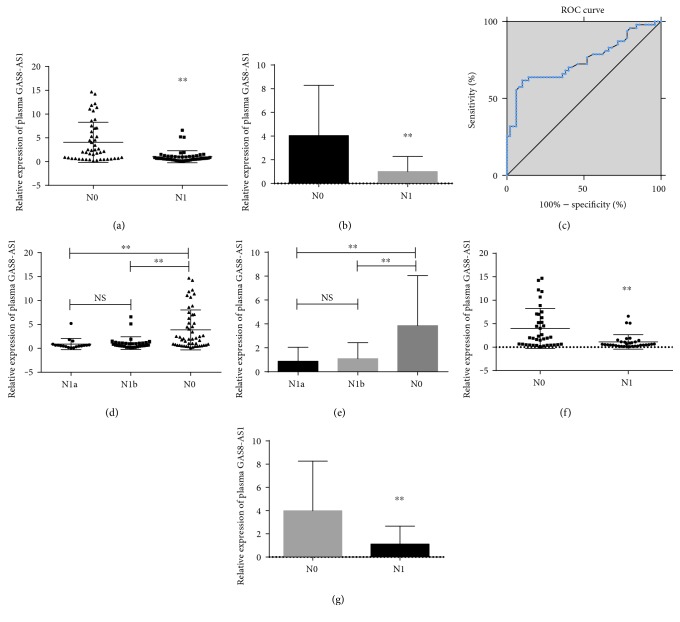
RT-qPCR and ROC curve analysis for GAS8-AS1 as an LNM prediction biomarker. (a) Scatter plots and (b) bar charts of plasma GAS8-AS1 levels from papillary thyroid cancer (PTC) patients with (N1, *n* = 50) and without (N0, *n* = 47) cervical lymph node metastasis (LNM). (c) ROC to evaluate the diagnostic performance of GAS8-AS1 to discriminate N1 from N0. (d) Scatter plots and (e) bar charts of plasma GAS8-AS1 levels from PTC patients with N1a stage (*n* = 18), N1b (*n* = 32), and N0 (*n* = 47). (f) Scatter plots and (g) bar charts of plasma GAS8-AS1 levels from papillary thyroid microcarcinoma (PTMC) patients with (N1, *n* = 34) and without (N0, *n* = 34) cervical lymph node metastasis (^∗∗^*P* < 0.001).

We confirmed that plasma lncRNA GAS8-AS1 expression was lower in the thyroid with LNM than in those without metastasis (*P* < 0.001) as well as in PTMCs (*P* < 0.001) (Figures 2(a), 2(b), 2(f), and 2(g)). Further multivariate analysis showed that a reduced GAS8-AS1 level in the plasma was associated with LNM (Table 1). An ROC was generated to evaluate the role of GAS8-AS1 in LNM prediction (Figure 2(c)). When the cutoff value was 1.58, the AUC was 0.746 (95%CI=0.645–0.847, *P* < 0.001, sensitivity was 61.70%, and specificity was 90.00%). A total of 51.55% of patients suffered from LNM, 92.00% of whom had central cervical LNM (level IV). In addition, a reduced GAS8-AS1 level was found in N1a and N1b (according to the AJCC 7th edition/TNM Classification System for Differentiated Thyroid Carcinoma) (*P* < 0.001) (Figures 2(d) and 2(e)), while there were no differences between cervical departments (N1a and N1b).

## 4. Discussion

PTC is the most common type of thyroid tumor. Although most patients with PTC have good prognosis after surgical resection in combination with radioiodine and levothyroxine treatment, metastasis and recurrence sometimes happen [10]. Many patients mainly die because of insufficient specific diagnostic biomarkers and therapeutic strategies [11]. Thus, successful prevention and treatment of PTC require a thorough understanding of its biological process and novel diagnostics and prognostic biomarkers.

lncRNAs are transcribed RNA molecules of more than 200 nucleotides that cannot code proteins [12]. Many studies have demonstrated the diverse cellular functions of lncRNAs including cell proliferation, cell differentiation, cell apoptosis, and carcinogenesis [13]. Several studies demonstrated that circulating lncRNAs are potential diagnostic biomarkers for several types of cancer [14]. For example, prostate cancer gene 3 (PCA3) is the first prominent biomarker of lncRNAs. Detection of PCA3 in urine outperformed PSA in prostate cancer diagnosis [15, 16]. Circulating lncRNA HULC (highly upregulated in liver cancer) and Linc00152 were significantly upregulated in the plasma of patients with hepatocellular carcinoma, and a combination of the two lncRNAs improved the diagnostic accuracy of alpha-fetoprotein, a biomarker for hepatocellular carcinoma diagnosis for patients [17]. Several studies [18–20] confirmed aberrant expression of circulating lncRNA H19 in the plasma of patients with gastric cancer compared with healthy controls.

With respect to PTC, it was reported that lncRNA GAS8-AS1 c.713A>G/714T>C nucleotide substitution induced a significant reduction in the RNA profile and a reduced lncRNA GAS8-AS1 level was observed in PTC tissues without mutation compared to those in adjacent thyroid tissues. Ectopically expressed GAS8-AS1 significantly suppressed cell viability in multiple thyroid cancer cell lines, while depletion of endogenous GAS8-AS1 expression markedly increased PTC cell proliferation [9]. In this study, we found that the expression of GAS8-AS1 was lower in the plasma compared with that observed in NG. We demonstrated that plasma GAS8-AS1 expression was dramatically lower in patients with primary PTC than in those with benign nodule controls. We also demonstrated that plasma GAS8-AS1 was lower in patients with LNM, but not those at advanced TNM stages. The same results were observed in papillary thyroid microcarcinomas. In addition, GAS8-AS1 was an independent risk factor of LNM in multivariable analysis, and the AUC for GAS8-AS1 to discriminate LNM was up to 0.746. All findings indicate that GAS8-AS1 can be used as a biomarker for LNM prediction. In many patients with PTC, LNM cannot be detected on preoperative imaging [21] or by inspection during surgery [22]. Therefore, whether to conduct prophylactic central-compartment neck dissection remains a difficult choice for physicians [23–25]. Plasma GAS8-AS1 is a potential maker that can be used in the decision-making process.

One limitation of the present study is that additional structural or functional studies are needed to evaluate the role of GAS8-AS1 in the carcinogenesis and aggressiveness of PTC. We also plan to investigate the predictive value of GAS8-AS1 in patients with PTC in a larger prospective cohort study.

## 5. Conclusions

In conclusion, our results suggest that decreased plasma GAS8-AS1 is a potential biomarker for PTC detection. In addition, plasma GAS8-AS1 may predict tumor aggression in patients with PTC.

## Figures and Tables

**Table 1:** Multivariate analysis of the association between papillary thyroid cancer (PTC)/lymph node metastasis (LNM) and reduced lncRNA GAS8-AS1 expression.

	lncRNAGAS8-AS1		
	OR	95% CI	*P* value
PTC diagnosis	0.891	0.817–0.972	0.010^∗^
Model 1			
Lymph node metastasis			
Model 1	0.608	0.466–0.792	<0.001^∗∗^
Model 2	0.521	0.336–0.807	0.004^∗^

Model 1, adjusted for sex and age. Model 2, adjusted for sex, age, TSH before surgery, tumor size, extrathyroidal extension, multifocality, nodular goiter, and Hashimoto thyroiditis. ^∗^*P* < 0.05 and ^∗∗^*P* < 0.001.

**Table 2:** Correlation between lncRNA GAS8-AS1 and clinic-pathological characteristics in all patients with papillary thyroid cancer (PTC).

Characteristics	lncRNA GAS8-AS1		*χ* ^2^ value	*P* value
	Low (%) (*n* = 49)	High (%) (*n* = 48)		
Sex				
Male	18 (36.73)	8 (16.67)	4.977	0.038^∗^
Female	31 (63.27)	40 (83.33)		
Age (years)				
<45	23 (46.94)	29 (60.42)	1.771	0.224
≥45	26 (53.06)	19 (39.58)		
Extrathyroidal extension				
Yes	27 (55.10)	23 (47.92)	0.684	0.535
No	20 (40.82)	34 (70.83)		
Tumor size (cm)				
≤1	32 (65.31)	36 (75.00)	1.087	0.376
>1	17 (34.69)	12 (25.00)		
Lymph node metastasis				
Yes	16 (32.65)	31 (64.58)	9.898	0.002^∗^
No	33 (67.35)	17 (35.42)		
TNM staging				
I-II	36 (73.47)	41 (85.42)	2.115	0.146
III-IV	13 (26.53)	7 (14.58)		
Multifocality				
Yes	15 (30.61)	16 (33.33)	0.022	1.000
No	33 (67.35)	33 (68.75)		
Nodular goiter				
Yes	29 (59.18)	19 (39.58)	3.726	0.068
No	20 (40.82)	29 (60.42)		
Hashimoto thyroiditis				
Yes	8 (16.32)	9 (18.75)	0.099	0.795
No	41 (83.67)	39 (81.25)		

^∗^ *P* < 0.05, chi-squared test *P* value.

**Table 3:** Correlation between lncRNA GAS8-AS1 and clinic-pathological characteristics in all patients with papillary thyroid microcarcinoma (PTMC).

Characteristics	lncRNA GAS8-AS1		*χ* ^2^ value	*P* value
	Low (%) (*n* = 34)	High (%) (*n* = 34)		
Sex				
Male	14 (41.18)	5 (14.71)	5.916	0.029^∗^
Female	20 (58.82)	29 (85.29)		
Age (years)				
<45	15 (44.12)	15 (44.12)	0.000	1.000
≥45	19 (55.88)	19 (55.88)		
Extrathyroidal extension				
Yes	11 (32.35)	18 (52.94)	2.000	0.213
No	20 (58.82)	16 (47.06)		
Lymph node metastasis				
Yes	23 (67.65)	11 (32.35)	8.471	0.007^∗^
No	11 (32.35)	23 (67.65)		
TNM staging				
I-II	6 (17.65)	4 (11.76)	0.469	0.734
III-IV	28 (82.35)	30 (88.24)		
Multifocality				
Yes	13 (38.24)	11 (32.35)	0.258	0.800
No	21 (61.76)	23 (67.65)		
Nodular goiter				
Yes	18 (52.94)	14 (41.18)	0.944	0.466
No	16 (47.06)	20 (58.82)		
Hashimoto thyroiditis				
Yes	8 (23.53)	9 (26.47)	0.078	1.000
No	26 (76.47)	25 (73.53)		

^∗^ *P* < 0.05, chi-squared test *P* value.
